# Moderating Effects of Transformational Leadership, Affective Commitment, Job Performance, and Job Insecurity

**DOI:** 10.3389/fpsyg.2022.847147

**Published:** 2022-05-09

**Authors:** Hui Shao, Hai Fu, Yuemeng Ge, Weichen Jia, Zhi Li, Junwei Wang

**Affiliations:** ^1^School of Humanities and Communication, Ningbo University, Ningbo, China; ^2^School of Liberal Arts, Nantong University, Nantong, China; ^3^Faculty of International Tourism and Management, City University of Macau, Taipa, Macao SAR, China; ^4^School of Media and Law, NingboTech University, Ningbo, China

**Keywords:** transformational leadership, job performance, affective commitment, job insecurity, emotional commitment

## Abstract

This work explored the mediating effects of affective commitment on transformational leadership and job performance and job insecurity on transformational leadership and affective commitment. Meanwhile, the inter-relationships between the four verified the mediating effect of affective commitment, including job insecurity. The results were as follows: (1) transformational leadership and job performance were positively related. (2) Transformational leadership was proportional to an emotional commitment. (3) The affective commitment had a positive impact on job performance. (4) Transformational leadership indirectly positively affected job performance through the intermediary effect of affective commitment. (5) Transformational leadership regulated affective commitment through job insecurity. The more job security employees have, the higher the impact of transformational leadership on affective commitment; the lower the contrary.

## Introduction

In the twenty-first century, organizations are facing enormous challenges in terms of ecological environment, scientific and technological R&D, and economic and social changes, especially competition and reform. Leaders are essential to the organization, so they face tough challenges, guiding employees to achieve their goals by encouraging morale. They affect all levels of the organization, including the direction of organizational development, culture, atmosphere, and employee identification. Therefore, scholars in different periods have different definitions of the traits of leaders that promote the development of enterprises.

This work studied the effect of transformational leadership on job performance with affective commitment and job insecurity as moderating variables. The objectives were as follows: (1) the impact of transformational leadership style on employees’ job performance; (2) the impact of transformational leadership behavior on employees’ affective commitment; and (3) the indirect impact of job insecurity on affective commitment through transformational leadership behavior.

## Literature Review

### Transformational Leadership

Lyubykh et al. (2022) divided transformational leadership into four dimensions: Idealized influence or charisma refers to leaders building good images through self-confidence to maintain employees’ confidence. These leaders who have substantial autonomy and conversion abilities can persuade employees or meet their expectations through observed their needs, desires, and values (Haider et al., 2019).

Individualized consideration refers to leaders inspiring employees’ maximum potential through employee care and humanized management to improve creativity and learning ability. Intellectual stimulation can improve employees’ problem-solving ability, which means leaders inspire employees to solve problems from multiple angles and objective standpoint. Inspirational motivation means leaders using symbols and affective requirements to enhance employees’ enthusiasm to achieve common goals (Yusuf et al., 2020).

In short, the organizational vision and shares mission that employees agree on can inspire their enthusiasm. Leaders reduce the workload of employees through creative work, such as vibrant slogans (Akkaya and Burcu, 2019). This work believed that transformational leadership could be viewed as the process of influence between people and the organization mobilization, which can transform the individual interests of employees into the common interests of the organization. In addition to reforming organizational culture and values, leaders shape a shared vision with employees, which can arouse the organization members to work for a common goal consciously.

### Affective Commitment

Affective commitment is also called attitudinal commitment. Khan et al. (2019) believed that commitment exceeds utilitarian values, which is consistent with organizational values as an affective orientation. However, Haider et al. (2019) believed that affective commitment is the employees’ dependence on the organization under the emotional belonging, which is the same as the organizational goals and values. Therefore, it is more lasting than the material transaction relationship (Haider et al., 2019).

Fadhel et al. (2019) defined affective commitment in organizational commitment research as employees’ emotional dependence, while in consumption as consumers’ desire to tie together with service providers. However, Kurtessis et al. (2017) believed that it is an effort between employees and organizations to maintain mutual relations and shared values and emotional belonging. Employees’ affective commitment is based on the social relationship between employees and organizations, which connects the two parties and describes the emotional motivation of the employees to maintain the current relationship (Kaur and Paruthi, 2019).

This work argued that affective commitment indicated that employees had an emotional attachment to the organization with the same goals and values. They helped each other achieve personal goals and values to get close to the organization. The stronger the affective commitment to the organization, the higher the willingness to stay.

### Job Performance

Afsar and Umrani (2020) proposed a performance model, including proficiency in specific and non-specific job tasks, written and verbal communication skills, degree of effort, personal self-discipline, promotion of team performance, supervision, leadership, and administrative management. Kalia and Bhardwaj (2019) divided job performance into the task and contextual performance based on organizational citizenship behavior.

Task performance refers to behaviors directly or indirectly related to work, such as workability and experience, and the performance of employees’ ability and proficiency in completing tasks. Meanwhile, it is related to the technical level of the organization, so production quality and profit productivity are used to measure employees’ task completion and goal achievement (Kleszczewska et al., 2019).

Contextual performance includes interpersonal and will motivational factors. Gaye and Cavide (2019) believed that it includes five aspects: The employees should (1) take the initiative to complete off-duty work; (2) be enthusiasm for work; (3) cooperate with others; (4) implement organizational regulations; and (5) support organizational goals. Therefore, according to job performance literature, this work studied job performance from the task and contextual performance.

### Job Insecurity

Block and Fisch (2006) divide job insecurity into quality and quantity. Quality refers to the threat of losing job characteristics or values, such as promotion, salary increase, and future career development, while quantity is the uncertainty of unemployment. Job insecurity is employees’ perception that their work is threatened without improving, which can be discussed from a multidimensional or global perspective:

1. Multidimensional dimension: Black and Hendy (2019) believed the severity of threat and powerlessness forms the job insecurity. The former refers to the degree to which employees lose their job characteristics or organize their work (Black and Hendy, 2019), while the latter refers to the ability to resist threats, including lack of security, blurred expectations, authoritative working environment, and rigid dismissal procedures.
2. Global dimension: The global analysis of Caplan (2010) believed that job insecurity refers to the threat caused by unemployment or job uncertainty, which is used in organizational crises when employees feel insecure about unemployment (Ahmet and Erhan, 2018). However, there is a difference between global and multidimensional views. The former is the threat of imminent unemployment, while the latter is the fear of possibility or unemployment.

## Research Hypothesis and Inference

**Figure 1:**
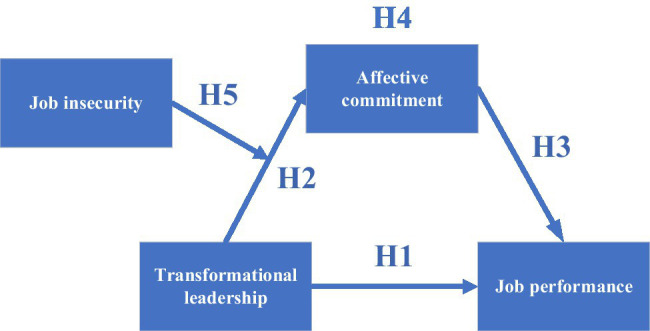
Theoretical model of the research.

Theoretical model of the research is shown in Figure 1.

### Transformational Leadership and Job Performance

Previous studies suggest that leader styles affect employee behaviors. Leaders’ behaviors affect the values of their subordinates by motivating them to shift from low-level needs to high-level during striving for organizational benefits (Clifton, 2019). However, different leadership styles have different incentive effects, wherein transformational leadership has the most substantial positive incentives with excellent performance level, which is related to the efforts of employees (Buchholz and Eichenseer, 2019).

Whether an employee can exceed the standard limit depends on driving factors such as the commitment to leadership, internal motivation, level of development, or sense of mission. However, transformational leadership must stimulate employees’ self-awareness and self-worth to achieve the highest performance (Boni and Sammut, 2019).

Indeed, research has found that every factor in transformational leadership can improve employees’ job performance, such as inspiring employees to complete challenging goals from a new perspective. Also, foreign scholars have demonstrated the relationship between transformational leadership and employee performance. To compare the vision and the performance of charm communication between transformational and charismatic leadership, Chunlin et al. (2019) simulated the completion of production tasks with 282 business-major students as experimental objects. Results showed that the quality and quantity of job performance have a positive impact on the implementation of the vision in the form of task clues (Chunlin et al., 2019).

Besides, scholars examined the impact of transformational leadership on the performance of business groups, finding that the motivational incentives were more significant than charismatic leadership in achieving the goals and performance. The results show that transformational leadership improves employees’ self-confidence and intrinsic value in completing tasks through charismatic behavior and encouragement, thereby improving job performance (Cameron et al., 2017).

This work believed that transformational leaders encouraged employees through transformational leadership behavior, which made employees rely on leaders and organizations. Then, employees’ hard work improved job performance. Therefore, hypothesis one has been proposed: Transformational leadership had a positive impact on job performance.

### Transformational Leadership and Affective Commitment

Transformational leadership achieves goals by trusting specific behaviors, such as allowing employees to participate in decision-making, sticking to their ideas, and fair discussion. If the employees can influence the decision-making in the process somewhat with the leaders, their cognition of trust is further enhanced (Ozgenel and Hidiroglu, 2019). The symbolic commitment of leaders will increase employees’ trust in the organization. Also, scholars have shown that the relationship between transformational leadership and emotional commitment is significant: transformational leadership can motivate employees to surpass personal interests to achieve team goals, while employees’ deep understanding of leaders will increase the affective commitment (Clifton, 2019).

Koh et al. (2018) found that principals’ transformational leadership is significantly related to teachers’ organizational commitment and students’ performance with teachers and principals of different schools in Singapore as the research objects. Besides, Edward and Kaban (2020) demonstrated the significant impact of transformational leadership on employees’ organizational commitment.

This work believed that leadership style was positively correlated with employees’ commitment. Transformational leadership encouraged employees to put organizational interests above personal by enhancing their self-confidence, thereby increasing employees’ affective commitment. Therefore, hypothesis two has been proposed: Transformational leadership has a positive impact on affective commitment (Youjeong et al., 2019).

### Affective Commitment and Job Performance

Affective commitment refers to an individual’s willingness to devote loyalty to the organization (Nivetha, 2018). As an internal driving force for employees to engage in beneficial behaviors for organizational goals, it allows them to trust the organization to achieve the value (Khan et al., 2019). Behaviors beneficial to the organization improve work performance. The affective commitment of employees indicates that the service provided by the organization meets their needs, which makes them improve job performance with loyalty.

Besides, domestic and foreign scholars have verified the relationship between affective commitment and job performance. Patnaik and Dubey (2019) pointed out that organizational commitment is related to job performance. Employees with higher organizational commitment have higher work engagement (Patnaik and Dubey, 2019). Khan et al. (2019) pointed out that employees with higher organizational commitment have higher job performance than the lower for higher resilience and adaptability.

This work believed that the affective commitment of leaders to employees was directly proportional to job performance. Employees distinguished themselves from others by working overtime voluntarily on rest days. They actively provided reasonable management suggestions as the masters of the organization. Also, their turnover rates were low, while the job performances were excellent. Therefore, hypothesis three has been proposed: Affective commitment has a positive impact on job performance.

### Transformational Leadership, Affective Commitment, and Job Performance

Employee’s affective commitment plays an essential role in transformational leadership and job performance (Pellegrini et al., 2020). Firstly, transformational leadership guides employees to make extra efforts by enhancing work value and employees’ confidence. Meanwhile, the trust felt by employees from the leader produces a higher affective commitment, with job performance exceeding expectations.

Secondly, transformational leadership affects job performance through affective commitment. Organizations increase employees’ trust in leaders by improving their affective commitment (Brimbahll, 2019). Besides, the confidence in the improvement of employee’s ability pays back to the organization, and their behaviors, including job performance, contribute to the development of the organization (Masaldzhiyska, 2019).

This work suggested that affective commitment was a bridge between transformational leadership and job performance. Therefore, hypothesis four has been proposed: Transformational leadership has a positive impact on job performance through affective commitment.

### Job Insecurity, Transformational Leadership, and Affective Commitment

Increased job insecurity in the organization causes negative emotions, which affects physical and mental health. Meanwhile, the reduction in affective commitment and loyalty leads to their departure (Bondarouk et al., 2018). Besides, the improvement of job insecurity makes employees question the necessity of staying in the organization. If there is no guarantee from the organization or leader, the trust of employees will be reduced, leading to resignation.

Clifton (2019) believed that the transformational leadership style has a positive impact on employees’ affective commitment. Employees feel insecure due to the loss of work rights, resulting in negative work, resignation, and resistance to changed organizational commitments (Basak, 2010).

Singh and Jaiswal (2016) proposed that the insecurity for job change directly reduces job value, satisfaction, and organizational commitment. This work believed that organizational management affected employees’ organizational commitment. Transformational leadership behaviors made employees experience high standards of ethics, which helped to achieve the goals by identifying with the organization with affective commitment.

Therefore, hypothesis five has been proposed: Transformational leadership has a positive impact on affective commitment through job insecurity. The lower the job insecurity, the higher the impact of transformational leadership on affective commitment.

## Instruments

### Overview

A questionnaire was constructed specifically for this study as there were no suitable instruments available. Four self-report questionnaires were integrated to measure the outcomes of this study. These questionnaires reference existing mature questionnaires and modified from prior studies to test reliability and validity. The pilot study was carried out among 150, who were not included in the formal study, to identify the possible errors of the questionnaire so as to improve the reliability (Cronbach’s alpha > 0.7) of the questionnaire.

The Cronbach’s α coefficient of this questionnaire survey is 0.755, exceeding 0.7, indicating its acceptable. Evaluated by a professional team, this questionnaire survey has a good content validity. For the questionnaire reference existing mature questionnaires, an exploratory factor analysis was tested to confirm the structure validity. The KMO = 0.978 > 0.5, and Barlett test of sphericity (*p* = 0.000 < 0.05), indicating this questionnaire is suitable for factor analysis. Rotated component matrix consists of four components, and the items in each component are as expected. The questionnaire survey is only for employees with the consent of participants. No privacy information of the participants is disclosed, which confronts the standards and requirements of scientific research.

The items in this questionnaire were written to reflect the transformational leadership, affective commitment, job performance, and job insecurity. All the statistical analysis has performed using IBM SPSS version 25.

### Transformational Leadership

#### Operational Definition

This work believed that transformational leadership was a process in which people interacted with each other to form mobilization forces. Leaders’ encouragement evoked employees’ self-confidence and self-awareness to consistent with organizational goals.

#### Measuring Method

This work adopted a scale translated from the MLQ of Clifton (2019) to measure transformational leadership style, which included charismatic leadership, spiritual inspiration, individualized care, and intellectual stimulation (Clifton, 2019). The scale with Cronbach’s α coefficient of 0.966 had 15 positive scoring questions, such as “The supervisor inspires me to participate in the company’s fare.”

#### Scoring Method

The scale used Likert’s Five Scaling Method to rank each question’s descriptive behavior from low to high as “never,” “rarely,” “sometimes,” “often,” and “always. “The high scores meant more transformational leadership behaviors.

### Affective Commitment

#### Operational Definition

Employees, who recognized organizational goals and values with emotional dependence, assisted in accomplishing organizational goals.

#### Measuring Method

This work used the scale of O’Reilly and Chatman (1986), with Cronbach’s α coefficient of 0.848, containing four questions, including “I am happy to join this organization.”

#### Scoring Method

The questionnaire was scored with Likert’s Five Scaling Method. The answers of all positive scoring questions ranged from “very disagree” to “very agree,” with high scores meaning more affective commitment of employees.

### Job Performance

#### Operational Definition

This work defined job performance as the behaviors and outcomes of employees to achieve organizational goals within a specified period, conducive to the improvement of organizational efficiency.

#### Measuring Method

The scale was revised from Baron and Kenny (1986) Job Performance Scale. Cronbach’s α coefficient of the scale was 0.947, with 11 questions, like “I often plan my work schedule,” which was divided into “task performance” and “peripheral performance” (Baron and Kenny, 1986).

#### Scoring Method

The questionnaire adopted Likert’s Five Scaling Method. All positive scoring questions were given 1–5 points for “very disagree” to “very agree,” with high scores representing high job performance.

### Job Insecurity

#### Operational Definition

This work believed that job insecurity was a subjective experience based on the overall evaluation of the existing working environment, which was the employees’ worry about the threat of the job.

#### Measuring Method

The scale was selected from Hellgren et al. (2010), with Cronbach’s α coefficient of 0.876. There were seven questions, where the 4th to 7th were reverse scoring questions (Hellgren et al., 2010), such as “I am worried that the organization will remove me from my post without my agreement.”

#### Scoring Method

The questionnaire used Likert’s Five Scaling Method to give a score of 1–5 for “strongly disagree” to “strongly agree.” In addition to the reverse scoring questions, the high scores represented high job insecurity.

## Research Results

This work adopted random sampling and questionnaires, which were distributed to employees in the service industry, manufacturing industry, high-tech industry, state organs, and the education industry. The places included Beijing, Jiangsu, Guangdong, Hubei, and Macao, which avoided the differences between the north and south and the first- and second-tier cities. The questionnaires were filled out online and distributed directly at the job site to save human costs and improve the effectiveness of recycling. Also, it adopted an anonymous method to reduce the worry about the filling consequences. Meanwhile, the respondents were informed that personal information was confidential before the test. This work analyzed the useful questionnaires recovered with statistical methods to verify the research goals and assumptions, which explored the relationship between transformational leadership, affective commitment, job performance, and job insecurity.

### Samples Description

This work collected sample data of employees from all walks of life by questionnaire. A total of 300 questionnaires were distributed, and 294 were recovered, including 24 invalid and 270 valid, with a recovery rate of 92%. The results showed that the demographic variables covered gender, age, marital status, education level, working experience, years of working with supervisors, job grades, and employers.

Table 1 shows that the majority of the retrieved data were women; 138 females accounted for 51%, while 132 males accounted for 49%. The overall average age was 33.7 years old, including 20–29 years old (42%), 30–39 (36%), 40–49 (14%), and over 50 (8%). In terms of marital status, 122 out of 270 were unmarried, accounting for 45%. As for education level, 40 people (15%) had high school education or below, 134 (50%) bachelors accounted the most, and 96 (35%) were masters or above.

**Table 1:** Summary of sample characteristics.

Project	Data category	Sample	Percentage (%)
Gender	Male	132	49
	Female	138	51
Age	20–29 Years old	113	42
	30–39 Years old	97	36
	40–49 Years old	38	14
	50 Years old above	22	8
Marital status	Unmarried	122	45
	Married	148	55
Education level	High school and below	40	15
	Undergraduate	134	50
	Master degree and above	96	35
Working experience	Under 1 Year	14	5
	1–10 Years	144	53
	11–20 Years	68	25
	20 Years and above	44	16
Years of working with supervisors	Under 1 year	19	7
	1–5 Years	148	55
	6–10 Years	81	30
	10 years and above	22	8
Job grades	Senior supervisors	50	19
	Junior supervisors	41	15
	Intermediate supervisors	42	15
	Technical personnel	27	10
	Grass-roots personnel	110	41
Employers	Service industry	90	33
	Manufacturing industry	27	10
	High-tech industry	36	13
	State organs	26	10
	Education industry	25	9
	Others	66	24

As for working experience, the majority of people working for 1–10 years accounted for 53, 25% for 11–20 years, 16% for 20 years above, and 5% for less than 1 year. For years of working with supervisors, 7% were under 1 year, and the most were 1–5 years with proportions of 55, 30% for 6–10 years, and 8% for more than 10 years.

The highest proportion of job grades in subjects was the grass-roots personnel (41%), followed by senior supervisors (19%), junior and intermediate supervisors (15%), and technical personnel (10%). The majority came from the service industry (33% of the total number), 10% from the manufacturing industry and the state organs, 13% from the high-tech industry, and 9% from the education industry.

### Variable Correlation Analysis

This work verified the correlation between demographic variables and transformational leadership, affective commitment, job insecurity, and performance with Kendall correlation coefficient. Table 2 shows the results:

1. Age s positively correlated with marital status and working experience, indicating that the number of married and working experience increases with age.
2. Transformational leadership was positively correlated with job performance and affective commitment, which was consistent with hypotheses one and two in the second chapter. Transformational leaders encouraged employees to produce high performance because their feelings of being focused on increased affective commitment.
3. Affective commitment is positively correlated with job performance, which is consistent with hypothesis three in the inference of the research hypothesis. High affective commitment indicates that the quality of service provided by the organization is good. The satisfaction of employees’ needs makes them loyal to the organization, which in turn improves job performance.
4. Affective commitment is negatively correlated with job insecurity, which reduces organizational commitment with the inability to the changing working environment without producing behaviors conducive to organizational development.

**Table 2:** Correlation analysis of variables.

	1	2	3	4	5	6	7	8
1. Gender	—							
2. Age	—	—						
3. Marital status	—	0.748^**^	—					
4. Working experience	—	0.813^**^	0.687^**^	—				
5. Transformational leadership	—	—	—	—	—			
6. Affective commitment	—	—	—	—	0.553^**^	—		
7. Job performance	—	—	—	—	−0.482^**^	−0.498^**^	—	
8. Job insecurity	—	—	—	—	0.265^**^	0.319^**^	−0.299^**^	—

^*^p < 0.05;

** *p* < 0.01;

^***^p < 0.001.

The correlation analysis indicates that the H1, H2, and H3 have been preliminarily verified.

### Hypothesis Testing

In this section, the relationships between transformational leadership, affective commitment, job insecurity, and performance were analyzed by hierarchical regression analysis based on the hypothesis of the second chapter.

Regression analysis assumes that there is no perfect exact relationship among exploratory variables. In regression analysis, when this assumption is violated, the problem of multicollinearity occurs. For the multicollinearity problem, a variance inflation factor (VIF) in regression analysis is used to assess multicollinearity, as shown in Table 3. The VIF identifies the strength of correlation among the variables. The independent variable is the transformational leadership, affective commitment, and job insecurity, and the dependent variable is job performance. In Table 3, each variables’ tolerance is in the range of [0.1, 1], and VIF values are both less than 10, indicating that there exists a low relationship among the exploratory variables; it is a type of low multicollinearity.

**Table 3:** Tolerance and VIF analysis.

Model		Unstandardized coefficients^a^		Standardized coefficients	*t*	*p*	Collinearity statistics	
		B	Std. error	Beta			Tolerance	VIF
1	(Constant)	5.52	0.084		65.48	0		
	Transformational leadership	−0.52	0.06	−0.563	−8.614	0	0.164	6.082
	Affective commitment	−0.317	0.063	−0.334	−5.047	0	0.161	6.227
	Job insecurity	−0.035	0.03	−0.033	−1.149	0.251	0.83	1.204

a Dependent variable: C job performance.

Significant at *p* < 0.05.

### Regression Analysis of Transformational Leadership and Job Performance

First, Model 1 in Table 4 analyzes the control variables, including gender, age, marital status, and working experience. Model 2 verifies that the main effect is transformational leadership. The results show that the impact of control variables on job performance positively correlates transformational leadership with job performance, with a standardized regression coefficient of 0.592 (*p* < 0.001), and durable explanatory power for job performance (=0.359), which verifies hypothesis one.

**Table 4:** Regression analysis of transformational leadership and job performance.

Job performance		
	Model 1	Model 2
**Control variables**		
Gender	0.046	−0.001
Age	−0.161	0.04
Marital status	0.09	0.033
Working experience	0.182	0.034
**Main effect**		
Transformational leadership		0.592^***^
*R* ^2^	0.015	0.359
Adjust *R*^2^	0	0.347
△*R*^2^	0.015	0.344

^*^p < 0.05;

^**^p < 0.01;

*** *p* < 0.001.

### Regression Analysis of Transformational Leadership and Affective Commitment

Model 1 in Table 5 analyzes the control variables, including gender, age, marital status, and working experience. Model 2 verifies that the main effect is transformational leadership. The results show that the impact of control variables on the affective commitment positively correlates transformational leadership with affective commitment, with a standardized regression coefficient of 0.686 (*p* < 0.001), and durable explanatory power for affective commitment (= 0.467), which verifies the second hypothesis.

**Table 5:** Regression analysis of transformational leadership and affective commitment.

Affective commitment		
	Model 1	Model 2
**Control variables**		
Gender	−0.014	−0.068
Age	−0.15	0.082
Marital status	0.084	0.018
Working experience	0.057	−0.114
**Main effect**		
Transformational leadership		0.686^***^
*R* ^2^	0.005	0.467
Adjust *R*^2^	−0.01	0.457
△*R*^2^	0.005	0.462

^*^p < 0.05;

^**^p < 0.01;

*** *p* < 0.001.

### Regression Analysis of Affective Commitment and Job Performance

Model 1 in Table 6 analyzes the control variables, including gender, age, marital status, and working experience. Model 2 verifies that the main effect is affective commitment. The analysis shows that the impact of control variables on job performance positively correlates affective commitment with job performance, with a standardized regression coefficient of 0.663 (*p* < 0.001), and durable explanatory power for job performance (=0.452), which verifies the third hypothesis.

**Table 6:** Regression analysis of affective commitment and job performance.

Job performance		
	Model 1	Model 2
**Control variables**		
Gender	0.046	0.055
Age	−0.161	−0.061
Marital status	0.09	0.035
Working experience	0.182	0.144
**Main effect**		
Affective commitment		0.663^***^
*R* ^2^	0.015	0.452
Adjust *R*^2^	0	0.442
△*R*^2^	0.015	0.438

^*^p < 0.05;

^**^p < 0.01;

*** *p* < 0.001.

### Regression Analysis of Transformational Leadership, Affective Commitment, and Job Performance

According to the mediating effect of James and Brett (1984), the independent and dependent variables should satisfy the three principles of the effect. This work verified the effect of the independent and the intermediate variables on the dependent variable. The standard regression coefficients were obvious, which satisfied the first two principles of the effect.

Model 1 in Table 7 analyzes the control variables, including gender, age, marital status, and working years. Model 2 verifies the main effect of transformational leadership, while Model 3 verifies transformational leadership and affective commitment, which are hierarchical regression analysis.

**Table 7:** Regression analysis of transformational leadership, affective commitment, and job performance.

Job performance			
	Model 1	Model 2	Model 3
**Control variables**			
Gender	0.046	−0.001	0.032
Age	−0.161	0.04	0
Marital status	0.09	0.033	0.024
Working experience	0.182	0.034	0.09
**Main effects**			
Transformational leadership		0.592^***^	0.256^***^
Affective commitment			0.490^***^
*R* ^2^	0.015	0.359	0.487
Adjust *R*^2^	0	0.347	0.475
△*R*^2^	0.015	0.344	0.128

^*^p < 0.05;

^**^p < 0.01;

*** *p* < 0.001.

The results show that after the influence of control variables on job performance, there is a positive correlation between transformational leadership and job performance in Model 2, with a standardized regression coefficient of 0.592 (*p* < 0.001). Model 3 adds affective commitment as an independent variable, the standardized regression coefficient of transformational leadership on job performance is reduced from 0.592 to 0.256, and the value of affective commitment on job performance is 0.490, with an apparent explanatory power (*p* < 0.001), which satisfies the third principle of James and Brett (1984) intermediary effect.

Meanwhile, the standardized regression coefficient of transformational leadership and affective commitment is 0.686, while the coefficient of affective commitment and job performance in Model 3 in Table 7 is 0.490. The multiplication of the two values plus the coefficient of transformational leadership and job performance of 0.256 is equal to the coefficient of transformational leadership and job performance of 0.592 in Model 2. The above proves that affective commitment has a partial mediating effect between transformational leadership and job performance.

### Moderating Effect of Job Insecurity in Transformational Leadership and Affective Commitment

Model 1 in Table 8 analyzes the control variables, including gender, age, marital status, working experience, and working experience with the supervisor. Model 2 verifies that the main effects are transformational leadership and job insecurity. The results show that the impact of control variables on affective commitment positively correlate transformational leadership with affective commitment, while job insecurity is negative. The standardized regression coefficients are 0.575 and −0.226, respectively, reaching a significant level (*p* < 0.001).

**Table 8:** Moderating effect of job insecurity in transformational leadership and affective commitment.

Affective commitment			
	Model 1	Model 2	Model 3
**Control variables**			
Gender	−0.014	−0.062	−0.062
Age	−0.15	0.063	0.079
Marital status	0.084	0.03	0.026
Working experience	0.057	−0.097	−0.107
**Main effects**			
Transformational leadership		0.575^***^	0.556^***^
Job insecurity		−0.226^***^	—
**Interaction terms**			
Transformational leadership × Job insecurity			−0.102^*^
*R* ^2^	0.005	0.506	0.516
Adjust *R*^2^	−0.01	0.495	0.503
△*R*^2^	0.005	0.501	0.01

* *p* < 0.05;

^**^p < 0.01;

*** *p* < 0.001.

Finally, Model 3 verifies the interaction effect. The results show that the interaction between transformational leadership and job insecurity reaches a significant level, with a standardized coefficient of −0.102 (*p* < 0.01) and a durable explanatory power for affective commitment (=0.516), which verifies Hypothesis 5.

**Figure 2:**
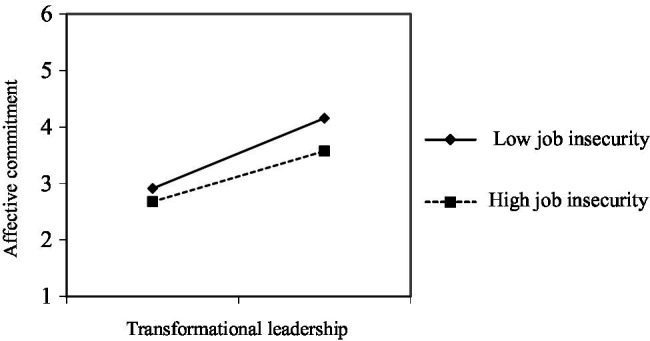
Interaction diagram.

To understand the direction of interaction and the relationship between employees’ different degrees of job insecurity, transformational leadership, and affective commitment, the interaction diagram in Figure 2 shows that job insecurity determines the influence of transformational leadership on affective commitment. It shows that for employees with high job insecurity, the positive influence of transformational leadership on affective commitment is lower than that with low insecurity. The result validates employees’ sense of job insecurity regulates the fifth hypothesis, namely the impact of transformational leadership on affective commitment.

## Conclusion

This work explored the relationship between transformational leadership and job performance, transformational leadership, and affective commitment, affective commitment and job performance, and the mediating effect of affective commitment and job insecurity.

First, the establishment of Hypothesis 1 proved that transformational leadership had a positive impact on job performance, which was the same as the research of Chunlin et al. (2019). It showed that in the interaction between transformational leaders and employees, the establishment of organizational vision encouraged subordinates to participate in organizational construction. In addition to completing the tasks within the role, they did more work outside the role conducive to organizational development, which improved job performance.

Second, hypothesis 2 confirmed the positive correlation between transformational leadership and affective commitment of subordinates, which was consistent with Koh et al. (2018) and Barling et al. (1996). Transformational leaders cared for the subordinates, which made them feel autonomy to solve problems.

Third, Hypothesis 3 believed that employees’ affective commitment had a positive relationship with job performance, which was consistent with Patnaik and Dubey (2019). When the affective commitment was high, the ability to withstand stress was high, which improved job performance.

Fourth, Hypothesis 4 verified the mediating effect of affective commitment, confirming that transformational leadership had a positive impact on job performance through affective commitment. Transformational leaders made employees feel affective commitment, which promoted employees to produce job performance that was beneficial to the organization, indicating the bridge role of affective commitment.

Fifth, Hypothesis 5 verified the moderating effect of job insecurity, indicating that employees’ job insecurity regulated the influence of transformational leadership on affective commitment. The lower the job insecurity, the higher the impact of transformational leadership on affective commitment; vice versa. Transformational leaders gave employees autonomy. This incentive gave employees (with job insecurity) free space to show excellent performance and more behaviors that were beneficial to the organization.

## Data Availability Statement

The raw data supporting the conclusions of this article will be made available by the authors, without undue reservation.

## Ethics Statement

The studies involving human participants were reviewed and approved by the NingboTech University Ethics Committee. The patients/participants provided their written informed consent to participate in this study. Written informed consent was obtained from the individual(s) for the publication of any potentially identifiable images or data included in this article.

## Author Contributions

All authors listed have made a substantial, direct, and intellectual contribution to the work and approved it for publication.

## Conflict of Interest

The authors declare that the research was conducted in the absence of any commercial or financial relationships that could be construed as a potential conflict of interest.

## Publisher’s Note

All claims expressed in this article are solely those of the authors and do not necessarily represent those of their affiliated organizations, or those of the publisher, the editors and the reviewers. Any product that may be evaluated in this article, or claim that may be made by its manufacturer, is not guaranteed or endorsed by the publisher.
